# Neuroexcitatory effects of morphine-3-glucuronide are dependent on Toll-like receptor 4 signaling

**DOI:** 10.1186/1742-2094-9-200

**Published:** 2012-08-16

**Authors:** Michael R Due, Andrew D Piekarz, Natalie Wilson, Polina Feldman, Matthew S Ripsch, Sherry Chavez, Hang Yin, Rajesh Khanna, Fletcher A White

**Affiliations:** 1 Department of Anesthesia, Indiana University School of Medicine, 950 Walnut St, R2, Indianapolis, IN, 46202, USA; 2 Department of Pharmacology, Indiana University School of Medicine, 950 Walnut St, R2, Indianapolis, IN, 46202, USA; 3 Medical Neuroscience Program, Indiana University School of Medicine, 950 Walnut St, R2, Indianapolis, IN, 46202, USA; 4 Department of Chemistry and Biochemistry, University of Colorado at Boulder, JSCBB A224, Boulder, CO, 80302, USA; 5 Department of Anesthesia, Stark Neurosciences Research Institute, Indiana University School of Medicine, 950 Walnut St, R2, Rm 427, Indianapolis, IN, 46202, USA

## Abstract

**Background:**

Multiple adverse events are associated with the use of morphine for the treatment of chronic non-cancer pain, including opioid-induced hyperalgesia (OIH). Mechanisms of OIH are independent of opioid tolerance and may involve the morphine metabolite morphine-3-glucuronide (M3G). M3G exhibits limited affinity for opioid receptors and no analgesic effect. Previous reports suggest that M3G can act via the Toll-like receptor 4 (TLR4)/myeloid differentiation protein-2 (MD-2) heterodimer in the central nervous system to elicit pain.

**Methods:**

Immunoblot and immunocytochemistry methods were used to characterize the protein expression of TLR4 present in lumbar dorsal root ganglion (DRG). Using *in vitro* intracellular calcium and current clamp techniques, we determined whether TLR4 activation as elicited by the prototypical agonists of TLR4, lipopolysaccharide (LPS) and M3G, contributed to changes in intracellular calcium and increased excitation. Rodents were also injected with M3G to determine the degree to which M3G-induced tactile hyperalgesia could be diminished using either a small molecule inhibitor of the MD-2/TLR4 complex in rats or TLR4 knockout mice. Whole cell voltage-clamp recordings were made from small- and medium-diameter DRG neurons (25 μm < DRG diameter <45 μm) for both control and M3G-treated neurons to determine the potential influence on voltage-gated sodium channels (NaVs).

**Results:**

We observed that TLR4 immunoreactivity was present in peptidergic and non-peptidergic sensory neurons in the DRG. Non-neuronal cells in the DRG lacked evidence of TLR4 expression. Approximately 15% of assayed small- and medium-diameter sensory neurons exhibited a change in intracellular calcium following LPS administration. Both nociceptive and non-nociceptive neurons were observed to respond, and approximately 40% of these cells were capsaicin-insensitive. Increased excitability observed in sensory neurons following LPS or M3G could be eliminated using Compound 15, a small molecule inhibitor of the TLR4/MD-2 complex. Likewise, systemic injection of M3G induced rapid tactile, but not thermal, nociceptive behavioral changes in the rat, which were prevented by pre-treating animals with Compound 15. Unlike TLR4 wild-type mice, TLR4 knockout mice did not exhibit M3G-induced hyperalgesia. As abnormal pain sensitivity is often associated with NaVs, we predicted that M3G acting via the MD-2/TLR4 complex may affect the density and gating of NaVs in sensory neurons. We show that M3G increases tetrodotoxin-sensitive and tetrodotoxin-resistant (NaV1.9) current densities.

**Conclusions:**

These outcomes provide evidence that M3G may play a role in OIH via the TLR4/MD-2 heterodimer complex and biophysical properties of tetrodotoxin-sensitive and tetrodotoxin-resistant NaV currents.

## Introduction

Opioids such as morphine have been and continue to be used for the treatment of chronic pain. Adverse events such as opioid-induced hyperalgesia (OIH) often complicate the clinical course of pain treatment and may be due to the morphine metabolite morphine-3-glucuronide (M3G). Morphine is metabolized primarily in the mammalian liver into two metabolites, M3G and morphine-6-glucouronide (M6G) via glucuronidation [1,2]. Approximately 44 to 55% of morphine is converted to M3G and 9 to 15% to M6G [3,4]. M6G is thought to be responsible for much of the pain-relieving effects of morphine by acting on opioid receptors [2,5]. Due to low affinity for opioid receptors, M3G has effectively no analgesic effect [6,7]. However, administration of M3G in rodents elicits myoclonus and stimulus-dependent hyperalgesia [8,9].

The mechanism or receptor by which M3G induces these excitatory effects is unknown. However, actions of M3G in the nervous system are thought to occur through the Toll-like receptor 4 (TLR4) and the accessory secreted glycoprotein myeloid differentiation protein-2 (MD-2) complex to elicit proinflammatory glial activation [9,10]. More importantly, TLR4 knockout mice exhibit a three-fold leftward shift in the systemic dose of morphine necessary for analgesia when compared with wild-type mice [10]. Further evidence to support M3G as a novel TLR4 agonist is needed - particularly evidence of a direct effect on states of neuronal excitation and/or nociceptive behavior.

Agonist-induced activation of the TLR4/MD-2 complex is not limited to cells of the immune system or glial cells since the prototypical ligand of TLR4/MD-2 complex, lipopolysaccharide (LPS), can elicit the release of the neuropeptide calcitonin gene-related peptide (CGRP) from cultured dorsal root ganglion (DRG) and trigeminal root ganglion (TRG) sensory neurons and sensitize the transient receptor potential cation channel subfamily V member 1 (TRPV1) [11,12]. Neuronal release of CGRP is likely due to a combination of LPS-evoked TRPV1 sensitization and inward ion current changes; however, it is unknown whether the novel TLR4 agonist M3G has direct effects on states of neuronal excitation and/or nociceptive behavior.

The aim of our investigation was to test whether M3G administration increases sensory neuron excitation and alters nociceptive behavior in rodents. The M3G-dependent depolarization of sensory neurons would imply that there may be a number of ion channels downstream of TLR4 activation which may contribute to changes in nociceptive behavior. Using current clamp and whole-cell voltage-clamp recordings, we found that M3G increased excitability in small- and medium-sized acutely dissociated sensory neurons and enhanced tetrodotoxin-sensitive (TTX-S) and tetrodotoxin-resistant (TTX-R) voltage-gated sodium channel (NaV) densities in sensory neurons. We also demonstrated that M3G acting through the neuronal TLR4/MD-2 complex is likely responsible for rapid induction of tactile, but not thermal, nociceptive behavior following systemic M3G administration.

## Materials and methods

### Animals

Pathogen-free, adult female Sprague–Dawley rats (150 to 200 g; Harlan Laboratories, Madison, WI, USA) and adult male C57BL/10ScNJ and C57BL/6 mice (20 to 25 g; Jackson Laboratory, Bar Harbor, ME, USA) were used for all experiments. C57BL/10ScNJ mice exhibit a homozygous deletion of 74 kb at the *Tlr4* locus. Mice and rats were housed in temperature (23 ± 3°C) and light (12 hour:12 hour light:dark cycle; lights on at 07.00) controlled rooms with standard rodent chow and water available *ad libitum*. These experiments were approved by the IACUC of Indiana University/Purdue University in Indianapolis. All procedures were conducted in accordance with the Guide for Care and Use of Laboratory Animals published by the National Institutes of Health and the ethical guidelines of the International Association for the Study of Pain. Animals were randomly assigned to treatment or control groups.

### Drugs

All drugs were freshly prepared in saline on the day of the experiment and administered by intraperitoneal (i.p.) injections. A TLR4 small molecule inhibitor (Compound 15) was synthesized as described in detail in [13]. A stock solution of lipopolysaccharide (LPS) was reconstituted in sterile 0.1% BSA/PBS to 5 mg/ml, and aliquots were stored at −20°C (Sigma-Aldrich, St Louis, MO, USA). The concentration used was 1 μg/mL. Morphine-3-β-D-glucuronide (M3G) was supplied by NIH/NIDA Drug Supply Program and utilized at a concentration (3 μM) that is significantly less than the dose necessary to elicit responses in rodent central nervous system neurons [14,15].

### Tissue processing and immunocytochemistry for neural tissue

Naïve rat lumbar DRG tissue was collected after animals were sacrificed and transcardially perfused with Zamboni fixative. Primary antiserum used for immunocytochemical procedures [16] was anti-TLR4 goat L14 extracellular monoclonal antibody (1:200 dilution; Santa Cruz Biotechnology Inc., Santa Cruz, CA, USA), monoclonal anti-NeuN, polyclonal anti-CGRP and IB4-FITC (Sigma-Aldrich). After incubation with primary antibodies, 14 μm thick tissue sections were incubated in secondary antibodies (anti-goat made in horse conjugated to CY3, Jackson ImmunoResearch, West Grove, PA, USA). Positive control immunocytochemistry staining for TLR4 was conducted in rat spleen sections. Specific labeling of white pulp was observed (data not shown).

### Cell counts

Images were taken with an intensified charged coupled device camera (CoolSnap HQ2, Photometrics, Tucson, AZ, USA coupled to a Nikon microscope (Nikon Eclipse Ti; Nikon Instruments Inc., Melville, NY, USA) using Nikon Elements Software (Nikon Instruments Inc.). TLR4 immunopositive cell counts were conducted using Image Pro Software (Media Cybernetics, Bethesda, MD, USA). TLR4 cell counts were taken from at least eight serial tissue sections per L5 ganglia (70 μm between sections) and combined to reach the total percentage of neurons.

### Tactile behavioral assessment

Von Frey filaments were used to test mechanical sensitivity before and after M3G and/or Compound 15 administration. Prior to initial von Frey tactile testing, all rodents were habituated to testing chambers for at least 2 days. Animals were tested for baseline responses at least twice before initiation of the injection paradigm using previously published methods [16]. Briefly, the rat was placed on a metal mesh floor and covered with a transparent plastic dome where the animal rested quietly after an initial few minutes of exploration.

Animals were habituated to this testing apparatus for 15 minutes a day, 2 days prior to pre-injection behavioral testing. Following acclimatization, each filament was applied to six spots spaced across the glabrous side of the hind paw; two distinct spots for the distribution of each nerve branch (saphenous, tibial and sural). Mechanical stimuli were applied with seven filaments, each differing in the bending force delivered (10, 20, 40, 60, 80, 100, and 120 mN), but each fitted with a flat tip and a fixed diameter of 0.2 mm. The force equivalence of mN to grams is 100 mN = 10.197 g. The filaments were tested in order of ascending force, with each filament delivered for 1 second in sequence from the 1^st^ to the 6^th^ spot alternately from one paw to the other. The interstimulus interval was 10 to 15 seconds. A cutoff value of 120 mN was used; animals that did not respond at 120 mN were assigned that value. Stimuli were applied randomly to left and right hind paws to determine the stimulus intensity threshold stiffness required to elicit a paw withdrawal response.

The incidence of foot withdrawal was expressed as a percentage of six applications of each filament as a function of force. A Hill equation was fitted to the function (Origin version 6.0, Microcal Software Northampton, MA USA) relating the percentage of indentations eliciting a withdrawal to the force of indentation. From this equation, the threshold force was obtained and defined as the force corresponding to a 50% withdrawal rate. Mouse behavior was conducted in a similar fashion using a probe fitted with a flat tip and a fixed diameter of 0.1 mm. However, mechanical stimuli were applied to only one location on the glabrous side of the hind paw. All behavioral testing was performed by laboratory assistants who were blinded to the experimental conditions and unfamiliar with the experimental aims.

### Thermal behavioral assessment

Thermal hyperalgesia was determined by measuring foot withdrawal latency and duration of the response to heat stimulation [16]. Each rat was placed in a box (22 x 12 x 12 cm) containing a smooth glass floor. A heat source (UgoBasile Plantar™ Analgesia Instrument, Trappe PA, USA) was focused on a portion of the hind paw, which is flush against the glass, and a radiant thermal stimulus was delivered to that site. The stimulus shuts off automatically when the hind paw moves (or after 20 seconds to prevent tissue damage). The intensity of the heat stimulus was constant throughout all experiments. A thermal stimulus was delivered six times to each hind paw at 5-minute intervals. The value for the response based on thermal latency and duration of paw withdrawal was obtained by averaging five of six measurements per animal. The baseline response for right and left hind paws were tested for 2 days prior to initiation of the injection paradigm.

### Immunoblot methodology

Fresh frozen L3-L6 DRGs and TRGs were homogenized in modified RIPA buffer with protease/phosphatase inhibitors (USBio, Swampscott, MA, USA). Samples (40 μg/lane) were resolved by 10% SDS-PAGE and transferred to a nitrocellulose membrane. After incubation in 10% non-fat milk blocking solution overnight at 4°C, the membrane was incubated with primary antisera for 1 hour (anti-TLR4 goat M16; 1:1,000; Santa Cruz Biotechnology Inc., Santa Cruz, CA, USA). The membrane was reprobed with a monoclonal anti-β actin (1:10,000; Sigma-Aldrich). Immunopositive bands were detected by enhanced chemiluminescence (ThermoScientific, Rockford Ill, USA) using donkey anti-goat (Santa Cruz Biotechnology Inc.) or rabbit anti-mouse (Jackson ImmunoResearch) horseradish peroxidase-conjugated secondary antibodies.

### Preparation of acutely dissociated dorsal root ganglion neurons

Lumbar DRGs were acutely dissociated using methods previously described [16]. Briefly, L1-L6 DRGs were removed from naïve animals. The DRGs were treated with collagenase A and collagenase D in HBSS for 20 minutes (1 mg/ml; Roche Applied Science, Indianapolis, IN, USA), followed by treatment with papain (30 units/ml, Worthington Biochemical, Lakewood, NJ, USA) in HBSS containing 0.5 mM EDTA and cysteine at 35°C. The cells were then dissociated via mechanical trituration in culture media containing 1 mg/ml BSA and trypsin inhibitor (1 mg/ml, Sigma-Aldrich). The culture media was DMEM, Ham’s F12 mixture, supplemented with 10% fetal bovine serum, penicillin and streptomycin (100 μg/ml and 100 U/ml) and N2 (Life Technologies, Carlsbad CA, USA). The cells were plated on coverslips coated with poly-L lysine and laminin (1 mg/ml) and incubated for 2 to 3 hours before additional culture media was added to the wells. The cells were then allowed to sit undisturbed for 12 to 15 hours to adhere at 37°C (with 5% CO_2_).

### Intracellular calcium imaging

Acute dissociation of lumbar DRG and intracellular calcium imaging was performed using methods previously described [16]. The dissociated DRG cells were loaded with fura-2 AM (3 μM, Molecular Probes/Invitrogen Corporation, Carlsbad, CA, USA) for 25 minutes at room temperature in a balanced sterile salt solution (BSS; NaCl (140 mM), Hepes (10 mM), CaCl_2_ (2 mM), MgCl_2_ (1 mM), glucose (10 mM), KCl (5 mM)). The cells were rinsed with the BSS and mounted onto a chamber that was placed onto the inverted microscope. Intracellular calcium was measured by digital video microfluorometry with an intensified CCD camera (CoolSnap HQ2, Photometrics) coupled to a Nikon microscope (Nikon Eclipse Ti) and Nikon Elements Software. Cells were illuminated with a Lamda DG-4 175 W xenon lamp (Sutter Instruments, Novato, CA, USA), and the excitation wavelengths of the fura-2 (340/380 nm) were selected by a filter changer (Sutter Instruments). Sterile solution was applied to cells prior to LPS application, and any cells that responded to buffer alone were not used in LPS responsive counts. Compounds were applied directly into the coverslip bathing solution. LPS (1 μg/mL) was applied first, after which capsaicin (3 nM; Sigma-Aldrich), high K^+^ (50 μM) and ATP (3 nM) were added. Only calcium imaging traces that reflected at least a 50% increase over baseline were included in the analysis. All data were analyzed by two independent analyzers and only responses that were in agreement between the two individuals were used in the responsive cell counts.

### Electrophysiology

Sharp-electrode intracellular recordings were obtained from 4 to 18 hours after acute dissociation of lumbar DRG as previously described [17]. Coverslips were transferred to a recording chamber that was mounted on the stage of an inverted microscope. The chamber was perfused with 2 mL bath solution containing: NaCl, 120 mM; KCl, 3 mM; CaCl_2_, 1 mM; MgCl_2_, 1 mM; Hepes, 10 mM; glucose, 10 mM; adjusted to pH 7.4 and osmolarity 300 mosM. The recordings were obtained at room temperature. Electrodes were filled with 1.0 M KCl (impedance: 40 to 80 MΩ) and positioned by a micromanipulator (Newport Corporation, Irvine, CA, USA). A −0.1 nA current injection was used to bridge-balance the electrode resistance. Prior to electrode impalement, the size of the soma to be recorded was classified by eye according to its diameter as small (≤30 μm), medium (31–45 μm) and large (≥45 μm). Electrophysiological recordings were performed with continuous current-clamp in bridge mode using an AxoClamp-2B amplifier, stored digitally via a Digidata 1322A interface, and analyzed offline with pClamp 9 software (Axon Instruments, Union City, CA, USA). Only neurons with resting membrane potential more negative than −45 mV were analyzed. Neuronal excitability of small and medium, dissociated DRG sensory neurons was measured by injecting 1-second current pulses into the soma every 30 seconds. Current was adjusted in order to elicit two to four APs per current injection under baseline conditions. Following three control current injections, LPS (1 μg/mL recording solution) or M3G (3 μM) was applied to the coverslip and current injections continued every 30 seconds. Neuronal excitability was measured as the number of APs elicited per current pulse before and after addition of LPS or M3G. An attempt to reverse changes in excitability generated by LPS and M3G was performed using the TLR4 inhibitor, Compound 15 [13]. Compound 15 (50 μM) was added following two experimental current pulses in the presence of LPS or M3G in experiments where these ligands increased neuronal excitability.

Whole cell voltage-clamp recordings were made from small- and medium-diameter DRG neurons (25 μm < DRG diameter <45 μm) for both control and M3G treated neurons using a HEKA EPC10 amplifier as previously described [18]. To ensure the fidelity of the voltage-clamp during data acquisition all recordings were made using an extracellular bath solution containing reduced sodium (70 mM). TTX-S current densities were estimated following *post hoc* subtraction of the slow-inactivating TTX-R current [18]. TTX-R current densities were measured from recordings obtained in the presence of 500 nM TTX. NaV1.8 currents were estimated from the current elicited from a 150 ms pulse to 0 mV from a holding potential of −100 mV and NaV1.9 currents were estimated as the current remaining during the last 10% of a 150 ms test pulse to −60 mV from a holding potential of −100 mV.

### Statistics

GraphPad Software (LaJolla, CA, USA) was used to determine the statistical significance. The statistical significance of differences between means was determined by Student’s *t*-test or a one-way analysis of variance (ANOVA) followed by *post hoc*, pair-wise comparisons (Bonferroni’s method). Logistic regression was used to determine differences in the percentages of different groups of cells.

## Results

### Toll-like receptor 4 present on sensory neurons

**Figure 1:**
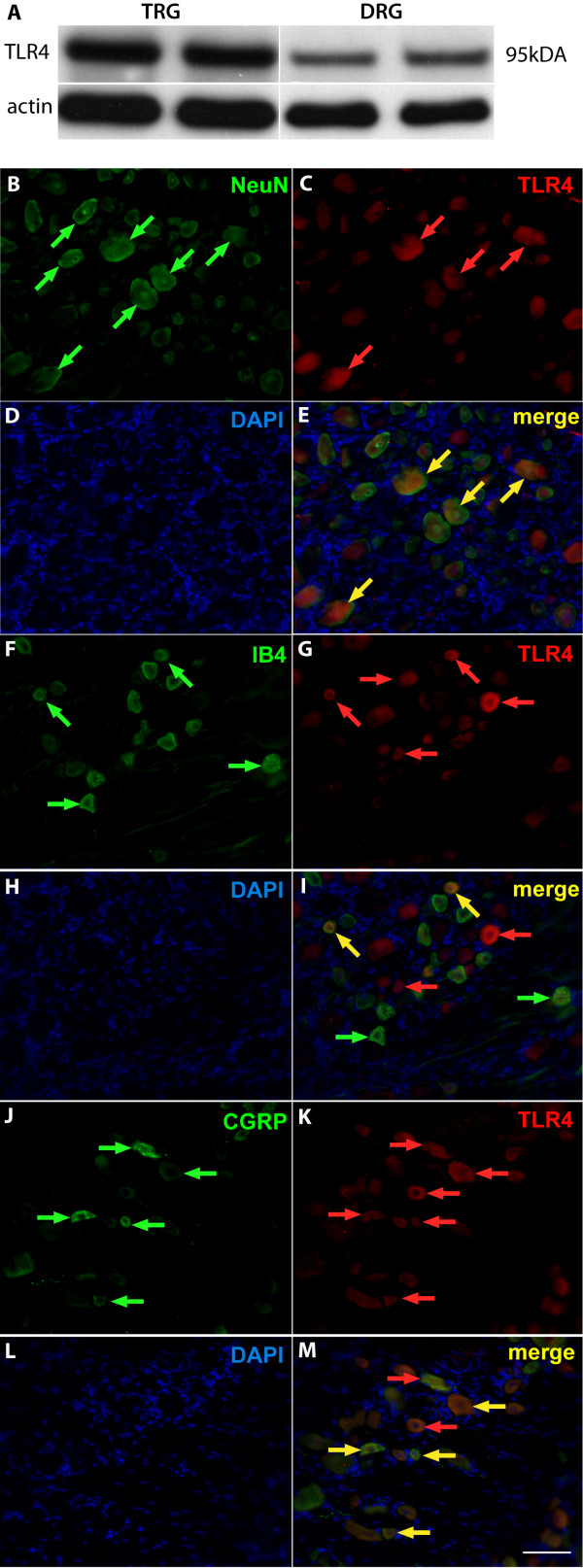
**Toll-like receptor 4 expression in sensory neurons.** (**A**) Western blot analysis of Toll-like receptor 4 (TLR4) isolated from trigeminal root ganglion (TRG) and lumbar L3-L6 dorsal root ganglion (DRG) lysates. (**B-M**) Immunofluorescent images of TLR4 in peptidergic and non-peptidergic DRG populations from sectioned naïve L4 or L5 DRGs. NeuN is a neuronal marker (**B**, green arrowheads), which co-labeled with TLR4 (**C**, red arrowheads). Nuclei are stained with DAPI (**D**, **H**, **L**, blue). Merged images demonstrate co-labeling of NeuN containing neurons with TLR4 (**E**, yellow arrowheads). Calcitonin gene-related peptide (CGRP) is a marker for peptidergic-containing sensory neurons (**F**, green arrowheads), which co-labeled with TLR4 (**G**, red arrowheads). Merged images demonstrate co-labeling of CGRP-containing neurons with TLR4 (**I**, yellow arrowheads). Isolectin B4 (IB4) is a marker for non-peptidergic sensory neurons (**J**, green arrowheads), which co-labeled with TLR4 (**K**, red arrowheads). Merged images show co-labeling of IB4 non-peptidergic sensory neurons with TLR4 (**M**, yellow arrowheads). Scale bar is 60 μm (**B-M**).

We first investigated the extent to which TLR4 protein expression is present in lumbar and trigeminal sensory ganglia (Figure 1A). TLR4 expression present within the DRG is localized exclusively in sensory neurons and averages 28 ± 5.6% of total sensory neurons per DRG assayed (1 L5 DRG per animal; n = 6 rodents). TLR4-immunopositive sensory neurons exhibit peptidergic and non-peptidergic neuronal phenotypes in the rat lumbar DRG (Figure 1)B-M.

### Lipopolysaccharide elicits intracellular calcium mobilization in dissociated sensory neurons

**Figure 2:**
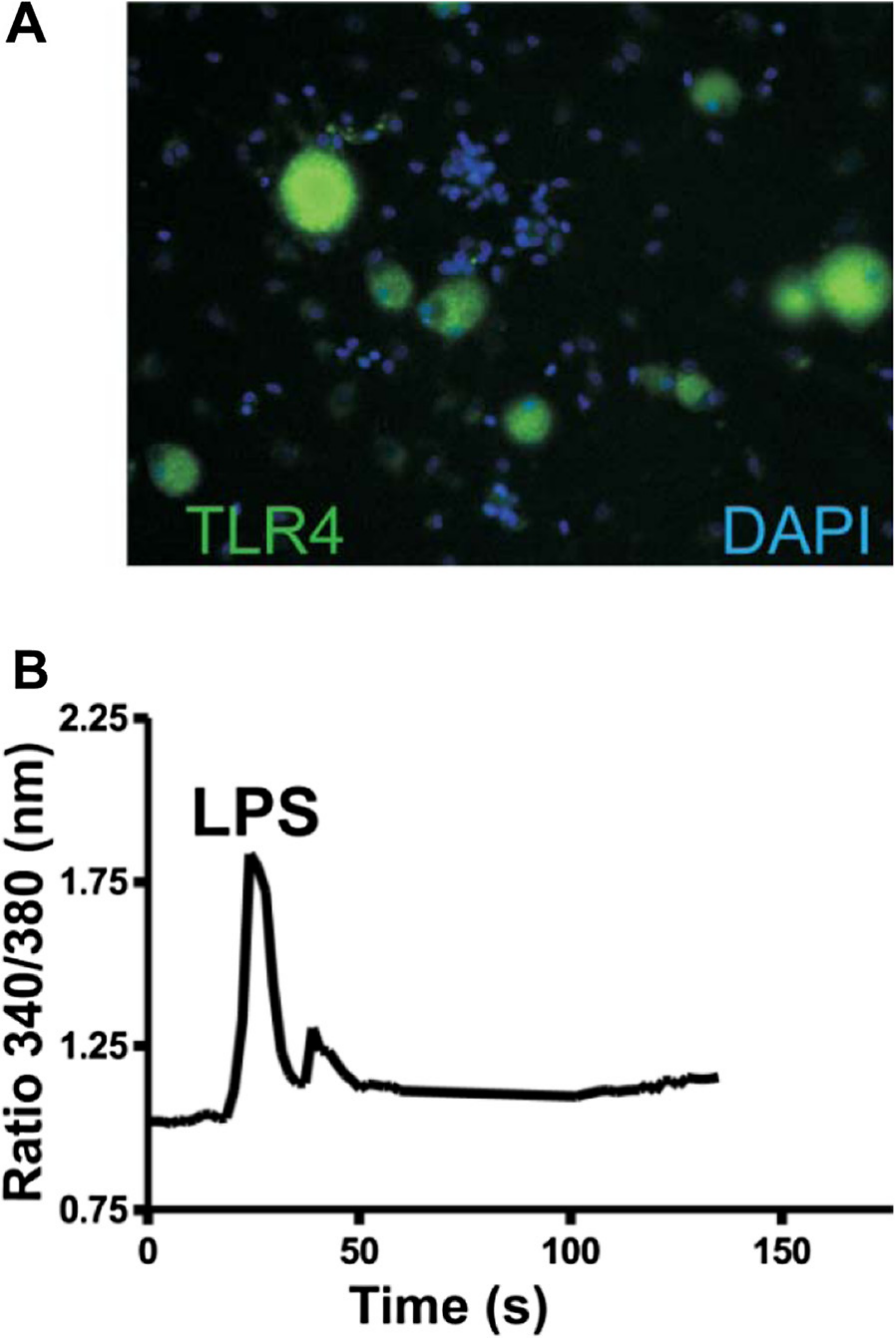
**Calcium imaging of functional Toll-like receptor 4 signaling in cultured dorsal root ganglion neurons.** (**A**) Toll-like receptor 4 (TLR4)-IR (green) is largely restricted to neurons and not non-neuronal cell types (blue, DAPI) in dissociated dorsal root ganglion (DRG) cultures. **(B)** Representative recording of a transient intracellular calcium increase in a dissociated DRG sensory neuron via lipopolysaccharide (LPS) administration (1 μg/mL).

Acute dissociation of DRG cells does not appear to alter the neuronal expression of TLR4 (Figure 2A). To functionally characterize neuronal TLR4 signaling we monitored intracellular calcium mobilization [Ca^2+^]_i_ following acute LPS administration (Figure 2B). The resultant cellular responses were categorized into three neuronal and non-neuronal cell types: non-capsaicin-sensitive neurons (high K and ATP responsive), capsaicin-sensitive neurons (capsaicin, high K, and ATP responsive), and glia (ATP responsive only). A moderate number of sensory neurons (approximately 14%) exhibited an LPS-induced [Ca^2+^]_i_ flux, of which 8.3% were capsaicin-sensitive (n = 85)and 5.9% were non-capsaicin-sensitive (n = 72). Likewise, LPS-responsive non-neuronal cells were limited in number (6.8%, n = 44) (Table 1).

**Table 1:** LPS response profile of acutely dissociated DRG cells from rats

Non-capsaicin-sensitive neuron	6% (5/85)
Capsaicin-sensitive neuron	8% (6/72)
Glia	7% (3/44)

### Lipopolysaccharide and morphine-3-glucuronide-induced sensory neuron excitation can be blocked by Toll-like receptor 4/myeloid differentiation protein-2 small molecule inhibitor

**Figure 3:**
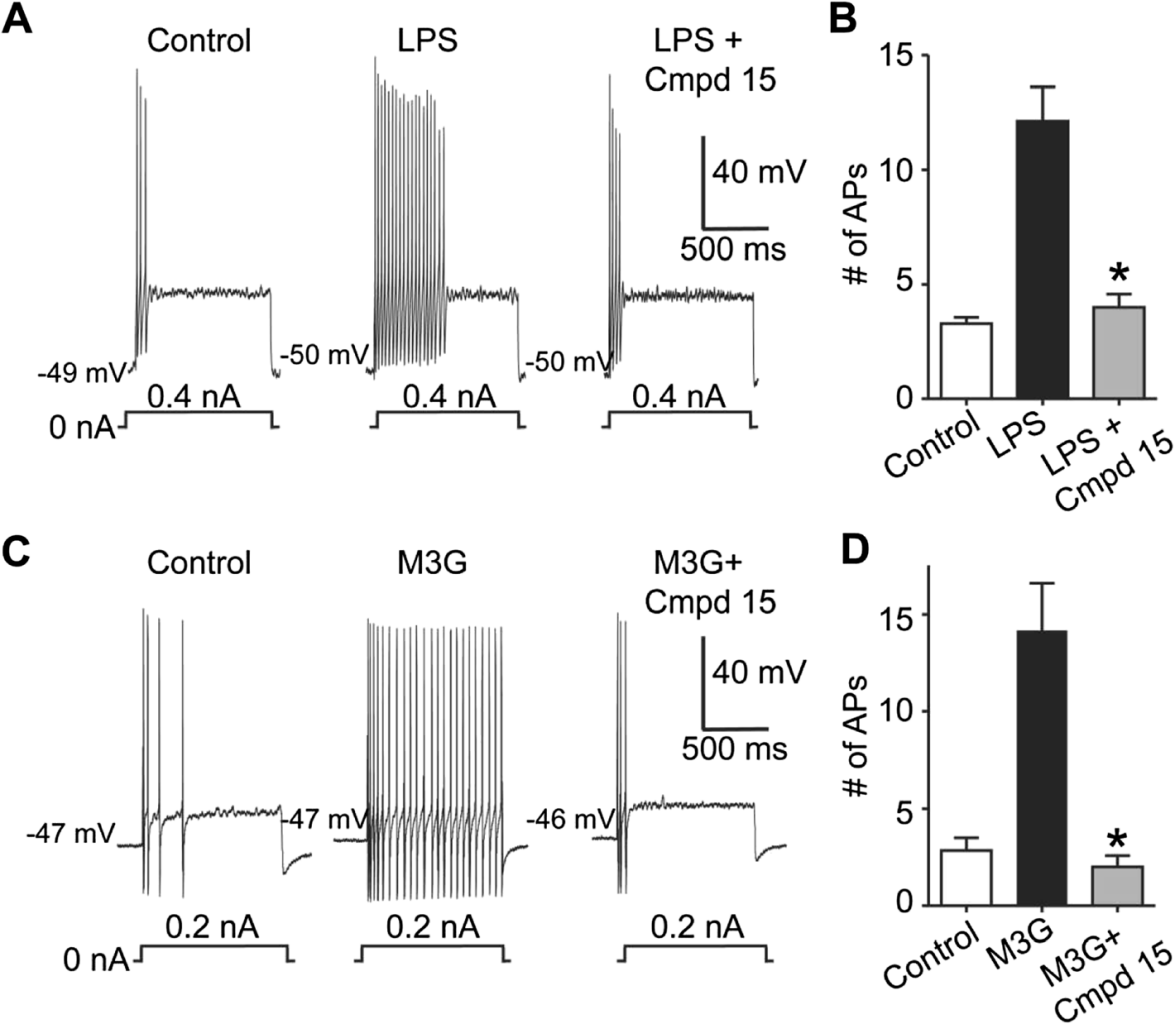
**Lipopolysaccharide and morphine-3-glucuronide increase the excitability of nociceptive dorsal root ganglion neurons.** Current clamp recordings were performed on small (≥30 μm) to medium (≥40 μm) dorsal root ganglion (DRG) neurons (L1-6) from naïve rats. Firing of two to four action potentials (APs) was elicited by a 1 second depolarizing current injection (ranging from 0.1 to 0.6 nA depending on the cell) every 30 seconds. (**A**) Representative recordings demonstrating that application of 2 μg/mL lipopolysaccharide (LPS) increases the number of elicited APs and Compound 15 can reverse this effect. (**B**) Group data demonstrating that LPS caused a significant increase in DRG AP firing that is reversed with Compound 15. (**C**) Representative recordings demonstrating that application of 3 μM morphine-3-glucuronide (M3G) increases the number of elicited APs and Compound 15 can reverse this effect. (**D**) Group data showing that M3G caused a significant increase in DRG AP firing that is reversed by Compound 15.

LPS has been shown to elicit an inward current in trigeminal sensory neurons [12] and enhance sensory neuron excitability in colonic nociceptive neurons [19]. Based on these observations, agonists of TLR4 should conceivably increase the excitability of sensory neurons in response to repeated current injection of the same amplitude. To determine the degree to which LPS-induced intracellular calcium mobilization reflects changes in sensory neuron excitation, we examined neuronal response using sharp electrodes in current clamp mode. Following repeated current pulse combined with LPS administration, we observed a significant increase in the excitability of small- to medium-diameter sensory neurons when compared to baseline levels (15.8% neurons responded, 3.2 ± 0.3 action potentials (APs) for control vs. 11.7 ± 1.7 APs for LPS, n = 38) (Figure 3). Representative recordings (Figure 3A) and grouped data (Figure 3B) demonstrate that the excitability of these neurons was significantly increased by LPS when compared with control levels. We utilized a previously reported small molecule inhibitor, Compound 15 [13,20], as a chemical probe to further investigate the molecular mechanism of M3G-induced neuron response. The ability of Compound 15 to disrupt the TLR4/MD-2 complex formation and selectively block TLR4 signal transduction without affecting other homologous TLR family proteins has been previously demonstrated [13]. Further, Compound 15 was screened against a panel of 12 representative kinases, showing negligible non-specific inhibitory effects [20]. Importantly, Compound 15 demonstrated high specificity and low toxicity both *in vitro* and *in vivo*[21], providing an excellent probing tool to study the TLR4-specific signal transduction. The specificity of this compound was utilized in our studies. Additionally, Compound 15 completely blocked the increased excitability of LPS suggesting that the observed increase in excitation is mediated through TLR4/MD-2 (100%, 4.5 ± 0.5 APs for LPS + Compound 15, n = 4; ANOVA, interaction F(2,18) = 21.29, *P* < 0.0001; Bonferroni multiple comparison test, *P < 0.05) (Figure 3A,B).

Given the ability of LPS to elicit increased excitability when combined with a depolarizing current injection in small- and medium-diameter sensory neurons, we tested whether M3G could produce similar effects in sensory neurons. The combination of repeated current pulse combined with M3G administration significantly increased the excitability of small- to medium-diameter sensory neurons when compared to baseline levels (19.6% neurons responded, 2.8 ± 0.7 APs for control vs. 14.2 ± 2.4 APs for M3G, n = 46) (Figure 3v). Subsequent treatment with the TLR4/MD-2 small molecule inhibitor Compound 15 completely blocked M3G-dependent excitability in sensory neurons (100%, 2.0 ± 0.6 APs for M3G + Compound 15, n = 4; ANOVA, interaction F(2,22) = 24.16, *P* < 0.0001; Bonferroni multiple comparison test, *P* < 0.05) (Figure 3C,D).

### Pretreatment of rodents with Toll-like receptor 4/myeloid differentiation protein-2 small molecule inhibitor prevents the rapid induction of tactile hyperalgesia due to systemic morphine-3-glucuronide administration

**Figure 4:**
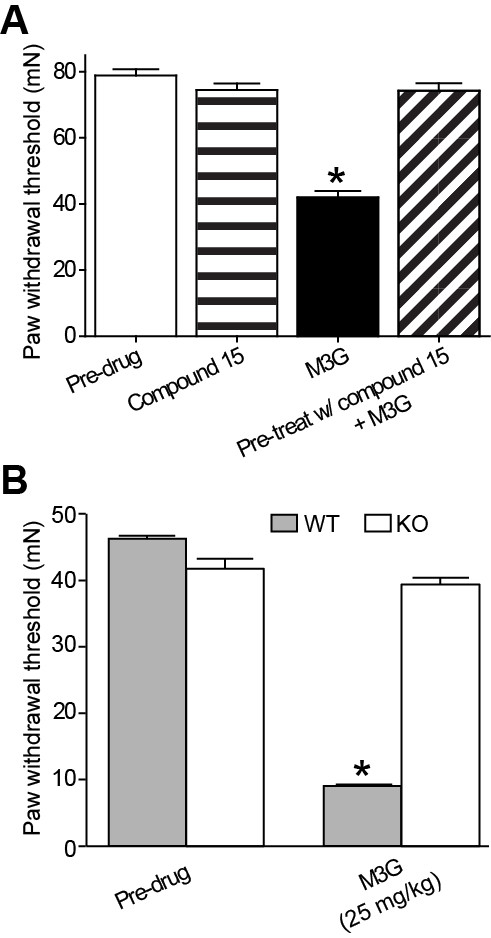
**Systemic morphine-3-glucuronide administration fails to elicit tactile hyperalgesia in rats pretreated with the small molecule inhibitor of Toll-like receptor 4/myeloid differentiation protein-2 complex, Compound 15, or in Toll-like receptor 4 knockout mice.** (**A**) Systemic morphine-3-glucuronide (M3G) (10 mg/kg, intraperitoneally) produced robust tactile hyperalgesia (n = 6, ANOVA, Bonferroni multiple comparison test, *P < 0.05), which could be prevented by pretreatment with Compound 15 (10 mg/kg, intraperitoneally, n = 6). (**B**) Shown is the force necessary to elicit paw withdrawal in Toll-like receptor 4 (*Tlr4*) wild-type and *Tlr4* knockout mice. Systemic M3G (25 mg/kg, intraperitoneally) produced robust tactile hyperalgesia in *Tlr4* wild-type mice (n = 6, *P < 0.05). *Tlr4* knockout mice failed to display a decrease in paw withdrawal force following systemic M3G (n = 6).

Direct administration of M3G into the central nervous system by intracerebroventricular or intrathecal routes produces a range of behaviors, including reductions in tail flick latencies, touch-evoked agitation and thermal hyperalgesia [8-10,22]. However, there is limited information regarding the effects of systemic M3G administration on noxious thermal or tactile stimulus-dependent behavioral outcomes. Though M3G (10 mg/kg, i.p.) failed to elicit changes in thermal latencies or duration (data not shown, n = 4; Student’s *t*-test, *P* > 0.05 for both), M3G produced significant reductions in the paw withdrawal threshold to tactile stimulus (Figure 4A; n = 6, *P* < 0.05). In contrast, paw withdrawal threshold to tactile stimulus in rodents pretreated with Compound 15 (10 mg/kg, i.p., 1 hour), a small molecule inhibitor of TLR4/MD-2 complex, prior to M3G administration (10 mg/kg, i.p.) did not differ from baseline thresholds or the combination of Compound 15 and vehicle (Figure 4A; n = 6, ANOVA, interaction F(3,20) = 69.33, *P* < 0.05; Bonferroni multiple comparison test, *P* < 0.05).

### Toll-like receptor 4 knockout fails to exhibit morphine-3-glucuronide-induced tactile hyperalgesia

To define whether M3G is dependent on the presence of a functional TLR4/MD-2 complex, we tested the degree to which M3G induces tactile hyperalgesia in a mouse line that exhibits a spontaneous mutation of the *Tlr4* gene [23]. Like rats treated with M3G, the *Tlr4* wild-type mice displayed a significant increase in tactile hyperalgesia (Figure 4B; n = 6, *t*-test, t = 71.55, df = 10, *P* < 0.05). However, *Tlr4* knockout mice failed to display tactile hyperalgesia following M3G administration (Figure 4B; n = 6, *t*-test, t = 0.278, df = 10, *P* > 0.05).

### Morphine-3-glucuronide increases sodium current density in both tetrodotoxin-sensitive and tetrodotoxin-resistant sodium currents

**Figure 5:**
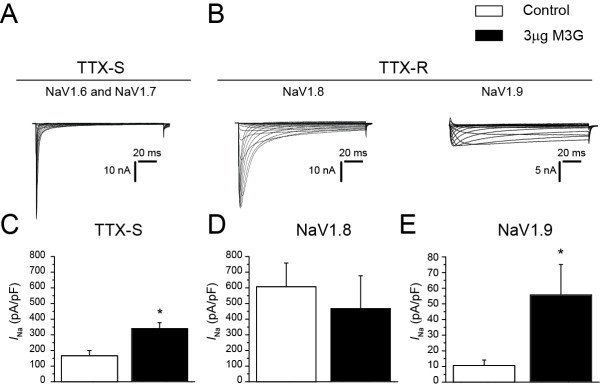
**Morphine-3-glucuronide-induced sensory neuron excitation is likely due to effects on voltage-gated sodium channels.** (**AB**) Representative current traces from acutely dissociated control dorsal root ganglion (DRG) neurons evoked by 200-ms steps in 5-mV increments applied from a holding potential of −100 mV. (**CE**) Peak current densities (pA/pF) of DRGs exposed to extracellular recording solution (control) or 3 μM morphine-3-glucuronide (M3G) for 5 minutes. Tetrodotoxin-sensitive (TTX-S) current densities were estimated using a pre-pulse inactivation protocol (500 ms pre-pulses) with a 0 mV test pulse as well as using *post hoc* kinetic subtraction [18]. Tetrodotoxin-resistant (TTX-R) current densities were made in the presence of 500 nM TTX to pharmacologically isolate the properties of voltage-gated sodium channel (NaV)1.8 and NaV1.9 currents. TTX-R NaV1.8 currents were estimated from the current elicited from a 150 ms pulse to 0 mV from a holding potential of −100 mV whereas TTX-R NaV1.9 currents were estimated as the current remaining during the last 15 ms of a 150 msec test pulse to −60 mV from a holding potential of −100 mV(*P < 0.05, versus control). Error bars indicate mean ± SE from at least 10 cells per condition. Small- and medium-diameter DRG neurons were used for these experiments.

TTX-S and TTX-R sodium currents have been shown to contribute to neuronal excitability in peripheral sensory neurons [24,25]. Here, we tested the possibility that TTX-S currents (Figure 5A) and TTX-R currents (NaV1.8 and NaV1.9; Figure 5B) could potentially be modulated by M3G and thus contribute to the increased hyperexcitability of sensory neurons in the presence of M3G. We compared untreated DRG neurons to neurons exposed acutely (approximately 5 minutes) to 3 μM M3G. We determined that TTX-S current density is increased approximately 2.5 times (Figure 5C, n = 12, *P* < 0.05) in the presence of M3G, and persistent TTX-R (NaV1.9) current density is increased approximately five times (Figure 5E, n = 12, *P* < 0.05) in the presence of M3G (*P* < 0.05). In contrast, NaV1.8 TTX-R current density is not affected by M3G (Figure 5D, n = 12, *P* > 0.05).

## Discussion

The production of the M3G metabolite following glucuronidation of morphine contributes to many off-target side effects of the opioid, including pain. Our results provide the first demonstration that M3G directly evokes sensory neuron excitability. The receptor responsible for this neuroexcitatory event appears to be the pattern recognition receptor, TLR4, as we have shown that treating sensory neurons with an inhibitor of the TLR4/MD-2 interaction, Compound 15, completely blocks the increased neuronal excitability evoked by M3G and effectively prevents systemic M3G-induced changes in tactile hyperalgesia. More importantly, whole-cell voltage-clamp recordings demonstrated that M3G increased TTX-S and TTX-R NaV densities in sensory neurons.

Little is known about the manner in which TLR4 contributes to pain. Broadly speaking, increased spinal microglial TLR4 activation correlates with both onset of behavioral hypersensitivity in rodent models of neuropathy and following administration of opioids [21,26]. However, the degree to which activation of TLR4 present on sensory neurons contributes to pain behavior is unknown. Clearly the ability of LPS administration to elicit intracellular calcium mobilization in both capsaicin- and non-capsaicin-sensitive neurons suggests that local administration of LPS to the plantar hind paw might produce thermal and mechanical sensitivities [27,28]. Likewise, the fact that M3G elicits neuronal excitation in both small- and medium-diameter sensory neurons might predict similar thermal- and tactile-dependent behavioral outcomes. Despite these expectations, systemic M3G administration evoked behavioral changes that were limited only to TLR4/MD-2-dependent mechanical sensitivities. This outcome suggests that short-term M3G exposure-mediated changes in tactile-mediated nociceptive behavior through TLR4 are independent of inflammation.

The apparent effects of M3G via TLR4/MD-2-mediated changes in tactile-dependent nociceptive behavior and non-capsaicin responsive cells may require unique ionic mechanisms such as modulation of voltage-gated sodium and/or potassium currents in addition to voltage-dependent calcium channels [29]. Here, we demonstrate that M3G increased TTX-S currents approximately 2.5 times. The TTX-S current in small-diameter neurons is most likely a combination of NaV1.6 and Nav1.7 [25]. These TTX-S channels can directly contribute to neuronal excitability [25], with NaV1.6 attributing to the resurgent sodium current and NaV1.7 attributing to amplifying subthreshold generator potentials by producing a prominent ramp current [30-32]. Interestingly, the LPS-induced activation of microglia appears to be dependent on TTX-S NaV1.6 channels [33].

The TTX-R sodium channel NaV1.9 has been shown to contribute to the genesis of heat and mechanical pain hypersensitivity [34]. In addition, NaV1.9 knockout mice have been shown to lack acute visceral hypersensitivity [35]. Given the five-fold increase in NaV1.9 current density, the action of M3G on this sodium channel isoform may be critical in the ability of M3G to generate hyperexcitability in sensory neurons. Supporting evidence includes observations that NaV1.9 enhances and prolongs the response to depolarizations that are subthreshold for AP electrogenesis [36]. NaV1.9 also functions to lower threshold for single AP and repetitive firing [37]. Thus, NaV1.9 may serve as an anion channel target for reducing M3G-induced pain sensitivity associated with morphine administration. Moreover, the ability of TLR4 signaling to modulate sodium channels elucidates a novel mechanism by which TLR4 agonists can influence neuronal states of excitation.

Mechanisms of excitation in primary sensory neurons via the TLR4/MD-2 complex remain elusive. However, due to the fact that IL-1β is known to increase the excitability of nociceptors via enhancing persistent TTX-R current [38,39], and IL-1β receptors signal through a common Toll/IL-1 receptor domain [40], it may come as no surprise that TLR4/MD-2 complex activation is capable of exciting neurons. Many other possible mechanisms exist which may also contribute to M3G-elicited neuronal excitation. It is possible that, in capsaicin-sensitive sensory neurons, LPS as a surrogate for M3G induces transactivation of TRPV1 via phospholipase C and results in the production of inositol 1,4,5-triphosphage and subsequent [Ca^2+^_i_ release cascade [41,42]. Subsequently, the increase in LPS-induced [Ca^2+^_i_ may depend on calcium influx via PKA (N-type calcium channels) or PKC pathways (non-N-type calcium channels) [12,19,43]. However, non-capsaicin-responsive cells would require other ionic mechanisms, such as modulation of voltage-gated sodium and/or potassium currents in addition to voltage-dependent calcium channels [29].

That the systemic M3G-induced behavioral outcome observed by our studies was limited to mechanical pain sensitivity may reflect unique capabilities of a TLR4/MD-2 dependent, capsaicin-insensitive sensory neuron population. Few experimental studies exist that delineate such a population with the exception of a subpopulation of unmyelinated polymodal nociceptive sensory neurons that express Mas-related G protein-coupled receptor member D (Mrgprd). Mrgprd appears to be selectively expressed in non-peptidergic nociceptors which when genetically ablated in the adult animal fail to respond to noxious mechanosensation whereas thermosensation is unaffected [44,45]. Whether the TLR4 agonist M3G is dependent on Mrgprd-positive sensory neurons for the changes in mechanical nociceptive behavior is unknown. Another subpopulation of sensory neurons that may be responsive to the administration of M3G is the non-peptidergic primary afferents that exhibit the delta opioid receptor and functionally contribute to mechanical pain [46].

In conclusion, TLR4 signaling via M3G within sensory neurons may provide a critical element for understanding the development of tolerance and paradoxical hyperalgesia that can occur following morphine-based therapies. The relationship between TLR4 expression in DRGs, tactile hyperalgesia and neuronal hyperexcitability via the morphine metabolite M3G may imply that TLR4-sensitized neurons may serve a vital signal to dampen the analgesic effects of morphine. Better understanding of these TLR4-mediated events in sensory neurons may provide the necessary framework for the design of agents that not only counteract deleterious opioid-induced cellular adaptations but also effectively reduce analgesic tolerance.

## Abbreviations

ANOVA: analysis of variance; AP: action potential; CGRP: calcitonin gene-related peptide; DRG: dorsal root ganglion; IL: interleukin; i.p.: intraperitoneal; LPS: lipopolysaccharide; M3G: morphine-3-glucuronide; M6G: morphine-6-glucouronide; MD: myeloid differentiation protein; Mrgprd: Mas-related G protein-coupled receptor member D; NaV: voltage-gated sodium channel; OIH: opioid-induced hyperalgesia; TLR: Toll-like receptor; TRG: trigeminal root ganglion; TRPV1: transient receptor potential cation channel subfamily V member 1; TTX-R: tetrodotoxin-resistant; TTX-S: tetrodotoxin-sensitive.

## Competing interests

The authors declare that they have no competing interests.

## Authors’ contributions

MRD performed the experiments, analyzed the data, and wrote the manuscript; AP performed the experiments, analyzed the data; NMW performed the experiments, analyzed the data; PF performed the experiments, analyzed the data; SC synthesized Compound 15; HY synthesized Compound 15; RK participated in the study design and data interpretation; FAW was the main investigator of this work, and was in charge of the study design, analysis and interpretation of results, and wrote the manuscript. All authors read and approved the final manuscript.
